# AHI-1: a novel signaling protein and potential therapeutic target in human leukemia and brain disorders

**DOI:** 10.18632/oncotarget.405

**Published:** 2011-12-30

**Authors:** Sharmin Esmailzadeh, Xiaoyan Jiang

**Affiliations:** ^1^ Terry Fox Laboratory, British Columbia Cancer Agency and Department of Medical Genetics, University of British Columbia, Vancouver, BC, Canada; ^2^ Department of Medicine, University of British Columbia, Vancouver, BC, Canada V5Z 1L3

**Keywords:** AHI-1, oncogene, CML, CTCL, AHI-1-BCR-ABL-JAK2 interaction complex, tyrosine kinase inhibitors, leukemic stem cells, TKI resistance, Joubert syndrome and susceptibility gene

## Abstract

Progress in the understanding of the molecular and cellular mechanisms of human cancer, including human leukemia and lymphomas, has been spurred by cloning of fusion genes created by chromosomal translocations or by retroviral insertional mutagenesis; a number of oncogenes and tumor suppressors involved in development of a number of malignancies have been identified in this manner. The *BCR-ABL* fusion gene, originating in a multipotent hematopoietic stem cell, is the molecular signature of chronic myeloid leukemia (CML). Discovery of this fusion gene has led to the development of one of the first successful targeted molecular therapies for cancer (Imatinib). It illustrates the advances that can result from an understanding of the molecular basis of disease. However, there still remain many as yet unidentified mutations that may influence the initiation or progression of human diseases. Thus, identification and characterization of the mechanism of action of genes that contribute to human diseases is an important and opportune area of current research. One promising candidate as a potential therapeutic target is *Abelson helper integration site-1*(*Ahi-1/AHI-1*) that was identified by retroviral insertional mutagenesis in murine models of leukemia/lymphomas and is highly elevated in certain human lymphoma and leukemia stem/progenitor cells. It encodes a unique protein with a SH3 domain, multiple SH3 binding sites and a WD40-repeat domain, suggesting that the normal protein has novel signaling activities. A new AHI-1-BCR-ABL-JAK2 interaction complex has recently been identified and this complex regulates transforming activities and drug resistance in CML stem/progenitor cells. Importantly, *AHI-1* has recently been identified as a susceptibility gene involved in a number of brain disorders, including Joubert syndrome. Therefore, understanding molecular functions of the *AHI-1* gene could lead to important and novel insights into disease processes involved in specific types of diseases. Ultimately, this knowledge will set the stage for translation into new and more effective diagnostic and treatment strategies.

## INTRODUCTION

Remarkable progress has been made in the last decade in the identification of oncogenes and tumor suppressors that are causative to the development of cancer. Many of these discoveries resulted from the cloning of fusion genes created by translocations that are characteristic of human leukemia and lymphomas [1-7]. The *BCR-ABL* fusion gene, associated with the development of chronic myeloid leukemia (CML) [1-6], is the prototype of such a discovery and has ultimately led to the development of one of the first targeted molecular therapies in cancer [4]. The revolution in the treatment of CML patients that has resulted from the specific and potent targeting of the BCR-ABL kinase with STI571/Gleevec/Imatinib Mesylate [8-10] serves as an important reminder of the advances that can come from an understanding of the molecular basis of disease. Retroviral insertional mutagenesis screens have been another powerful and complementary strategy in cancer gene discovery; a number of oncogenes and tumor suppressors that play crucial roles in development of human cancer and leukemia have been identified in this manner [11-15]. Recently, using retroviral-mediated insertional mutagenesis to identify genes that collaborate with oncogenes and tumor suppressors of the *Myc*, *p53*, *RB*, *Ras* and *ABL* pathways have resulted in a number of new targets for development of novel cancer therapeutics [13]. Nevertheless, specific molecular causes of many cancers remain unknown, along with the mutations responsible for a large proportion of human cancers. There is growing evidence that leukemogenesis, like the genesis of other malignancies, is a multi-step process requiring the accumulation of several mutations for the development of overt disease. Therefore, continued identification of new genes and characterization of the molecular basis of their transforming activity is critical to the future development of targeted cancer therapies that will be less toxic and more effective.

*Ahi-1 (Abelson helper integration site 1)* is a novel oncogene commonly activated by provirus insertional mutagenesis in *v-abl* and *myc*-induced murine leukemias and lymphomas [16]. It encodes a unique protein with a SH3 domain, multiple SH3 binding sites and a WD40-repeat domain, suggesting that the normal protein has novel signaling activities. *Ahi-1/AHI-1* transcript levels are normally down-regulated during both early murine and human hematopoietic cell differentiation and are highly deregulated in certain human leukemic cells, including leukemic stem cells from patients with chronic myeloid leukemia (CML) and leukemic Sezary cells in cutaneous T-cell lymphoma (CTCL) [17, 18]. Interestingly, overexpression of *Ahi-1* alone in primitive hematopoietic cells confers a proliferative advantage *in vitro* and induces a lethal leukemia *in vivo*; these effects can be enhanced by BCR-ABL, a fusion oncoprotein that plays a major role in the genesis of CML [19]. Importantly, a novel AHI-1-BCR-ABL-JAK2 interaction complex has recently been identified in CML cells, mediating these effects and playing a key role in mediation of tyrosine kinase inhibitor (TKI) response/resistance of primary CML stem/progenitor cells. These findings suggest that *AHI-1* could be a potential new therapeutic target in CML stem cells, a population highly resistant to current TKI therapy and thus causing disease relapse. Moreover, mutations in *AHI-1* have also been associated with Joubert syndrome, an autosomal recessive brain disorder [20-22]. Abnormal development and axonal decussation occur in individuals with point mutations in *AHI-1*, particularly within the WD40-repeat and SH3 domains [22]. Ahi-1 can also interact with Huntingtin-associated protein 1 (Hap1) to form a stable complex critical for neonatal development and involved in intracellular trafficking [23]. In addition, *AHI-1* isoforms and its mutations also underlie other diseases, including Joubert syndrome-associated nephronophthisis and autism, and metabolic syndromes, including type 2 diabetes [18, 19, 22, 24-27]. Therefore, it is likely that *AHI-1* mutations are critical in the development of diseases such as Joubert syndrome and specific types of human leukemia. Here we provide an extensive review of the molecular and cellular functions of AHI-1 and its interacting proteins in the regulation of normal and disease development since its identification as a cooperative oncogene in *v-abl*-induced murine leukemia in 2002 [16]. Also discussed are potential applications of targeting specific AHI-1 interaction complexes as a new therapeutic strategy for treatment of CML and brain disorders.

## IDENTIFICATION OF *Ahi-1/AHI-1* GENE BY PROVIRUS INSERTIONAL MUTAGENESIS IN MURINE LEUKEMIA AND LYMPHOMAS

### Identification of the *Ahi-1* gene in *v-abl-*induced mouse pre-B cell lymphoma

**Figure 1:**
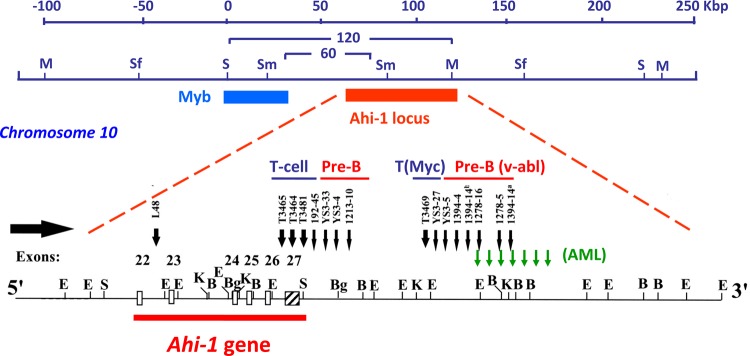
Identification of the *Ahi-1* gene by provirus insertional mutagenesis in various murine leukemias and lymphomas Schematic diagram of the positions of the Ahi-1 and Myb regions on mouse chromosome 10 within a 120 kbp genomic region. Structural organization of the proviruses integrated at the 3' end of the *Ahi-1* gene is indicated in *v-abl*-induced pre-B lymphomas, *Myc*-induced T-cell lymphomas and *Nf-1*-induced murine AML. The localization of the proviruses relative to the exons is shown.

The Ahi-1 (Abelson helper integration site-1) locus was initially identified as a common helper provirus integration site in 16% of Abelson murine leukemia virus (A-MuLV)-induced pre-B lymphomas (Figure 1) [28]. A-MuLV is a replication-defective murine retrovirus containing the *v-abl* oncogene which is responsible for its transforming potential [29-33] in murine models of leukemia and lymphomas. A-MuLV requires a non-defective helper MuLV virus to be able to replicate both *in vitro* and *in vivo* [34]. It was shown that the expression of *v-abl* is not sufficient to induce full malignant transformation in several mouse strains and that additional genetic events may be required [35, 36]. Using long-range restriction mapping, the Ahi-1 locus was mapped to a position ~35 kb downstream of the *c-myb* proto-oncogene on mouse chromosome 10 (Figure 1) [37]. Another MuLV provirus integration site (Mis-2) was also mapped to the same region, 160 kb downstream of c-myb and ~120 kb downstream of Ahi-1 [38]. It was observed that enhanced expression of *c-myb* in A-MuLV-induced pre-B-lymphomas harboring a provirus inserted within the Ahi-1 locus was not seen [37], suggesting that the locus contained another gene (potentially *Ahi-1* gene) whose dysregulation might be involved in regulation of the malignant transformation of hematopoietic cells. In addition, the Ahi-1 locus was also the target of provirus insertional mutagenesis in 14% of the *c-myc*-induced murine T cell leukemia (Figure 1) [39], 5% of the Moloney MuLV-induced rat thymomas [38], 11% of the *Hoxa9/Meis1*-induced murine acute myeloid leukemia [40] and in acute myeloid leukemias arising in *Nf1* heterozygous mice [41]. These findings suggest that the Ahi-1 locus is the target of provirus insertional mutagenesis and that its deregulation may contribute to multiple types of murine leukemia and lymphomas.

Subsequently, a new gene (*Ahi-1*) at the site targeted by the provirus insertional mutations within the Ahi-1 locus was then identified using an exon trapping method [16]. Most of the proviral insertions were localized to the 3' end of the *Ahi-1* gene in an inverse transcriptional orientation, primarily near and downstream of the last exon, although some were found within various introns (Figure 1). Cloning of the *Ahi-1* cDNA showed that it encodes a 1047 amino acid protein with a number of interesting domains characteristic of a novel signaling protein.

### The structure of Ahi-1/AHI-1 protein

**Figure 2:**
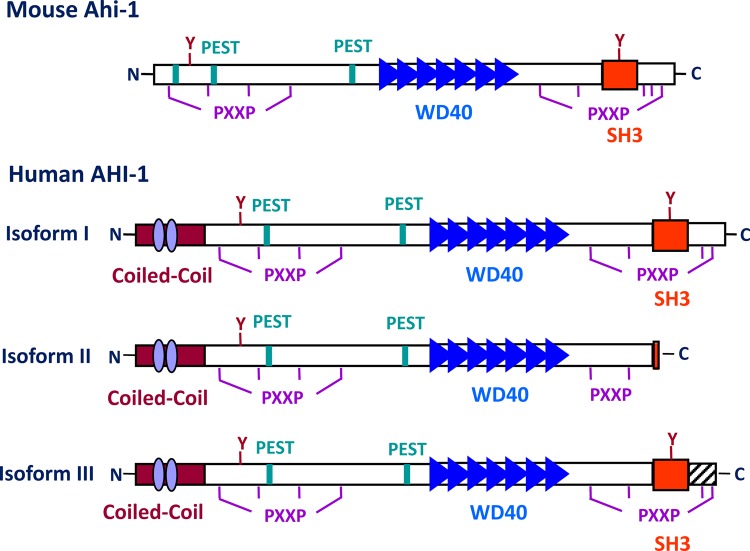
A schematic diagram of the mouse Ahi-1 and human AHI-1 (isoforms I-III) proteins A summary of the structural motifs present in the Ahi-1/AHI-1 proteins is shown, including: one SH3 domain (red box), seven WD40 repeats (blue triangles), proline-rich motifs (PXXP), PEST sequences (green boxes) and tyrosine kinase phosphorylation sites (Y). Human AHI-1 contains an additional coiled-coil domain which is absent from the mouse Ahi-1 (purple ovals). AHI-1 isoform II, the shortest isoform, lacks the SH3 domain and isoform III, which is still shorter than isoform I, contains additional coding sequences in its C-terminus which are absent from both isoform I and II.

The *AHI-1/Ahi-1* gene covers more than 200 kb in a region of human chromosome 6 and a minimum of 100 kb on mouse chromosome 10 (Figure 1). Mouse and human *Ahi-1/AHI-1* contain at least 27 and 33 exons, respectively [16]. The 1.3 kb region upstream of the ATG start codon of human *AHI-1* shows baseline promoter activity and contains two putative TATA boxes and several transcription factor binding sites, such as *Oct-1* and *c-fos* [42]. The *Ahi-1/AHI-1* gene is highly conserved in mammals and encodes a unique protein with a Src homology 3 (SH3) domain, multiple SH3 binding sites and multiple WD40-repeats, all known mediators of protein-protein interaction (Figure 2) [16, 43-46]. SH3 domains are often found in proteins containing SH2 domains and are known to bind to proline-rich motifs and to mediate specific protein-protein interactions [43, 44, 47]. The WD40-repeat domain was first identified in the β-subunits of G-proteins [45]. A large number of WD40-repeat-containing proteins have been identified and are known to be involved in aspects of cellular metabolism, including assembling and remodeling of chromosomal proteins [45, 46, 48-51] and regulating the mRNA processing body, including mRNA degradation. They have also been implicated in several inherited diseases, such as Cockayne syndrome, Triple-A syndrome and lissencephaly [51]. Interestingly, the Ahi-1/AHI-1 protein is the only protein thus far identified to contain both WD40 repeats and a SH3 domain. The SH3-binding sites were initially found to bind specifically to the SH3 domain of ABL [52]; many SH3 domain interacting proteins have been characterized as regulators of protein-protein interactions involved in signal transduction, cell cycle control and malignant transformation [43, 44, 46, 47]. *Ahi-1/AHI-1* also harbors two potential tyrosine phosphorylation sites, one within the SH3 domain. Phosphotyrosine motifs are known to bind specifically to SH2 domains of proteins [46]. In addition, several PEST sequences, known to mediate protein degradation [53, 54], are located in Ahi-1/AHI-1 protein. Moreover, the human AHI-1 contains a coiled-coil domain in its N-terminal region, also involved in protein-protein interactions [55], which is entirely absent in the mouse and rat Ahi-1 proteins (Figure 2) [16]. Thus Ahi-1 has multiple features of a unique adaptor protein regulating specific signaling pathways.

### *Ahi-1/AHI-1* expression in mouse brain development and hematopoiesis

Murine *Ahi-1* encodes two major RNA species (5.0 and 4.2 kb) and several shorter splice variants [16]. Both mouse and human *Ahi-1/AHI-1* are highly expressed in brain and testis and have lower expression in liver, lung, thymus, kidney and pancreas [16, 56]. We and others have recently demonstrated that *Ahi-1* transcripts are expressed at all stages of mouse embryo development, with increasing expression just prior to birth, suggesting that *Ahi-1* expression is developmentally regulated [16, 21, 56]. Mouse *Ahi-1* mRNA in the cerebellum has its highest expression at E18 and P5, whereas expression in the cerebral cortex appears maximally at E16 and E18 [21]. However, the expression of mouse Ahi-1 protein in the cerebellum is very low and is only restricted to Purkinje cells and cerebellar nuclei [56, 57]. Moreover, in adult human brain tissue, the highest expression of *AHI-1* at both RNA and protein levels has been detected in cerebellum and cerebral cortex [20, 56]. The different cerebellum expression of *Ahi-1/AHI-1* in mouse and human may be due to the additional coiled-coil domain present in the human *AHI-1* [56]. In addition, *Ahi-1* is also found to be abundant in the hypothalamus and amygdala, two important brain region whose dysfunction can lead to emotional and depression phenotypes [57, 58]. Interestingly, it has recently been reported that impaired Wnt-beta-catenin signaling that disrupts renal homeostasis leads to cystic kidney ciliopathy, as demonstrated in *Ahi-1* conditional mutant mice [59]. *Ahi-1* deficiency can also cause defective Wnt-dependent cerebellar midline fusion that is critical for the development of Joubert syndrome [60]. In addition, Ahi-1 is reported to directly interact with Hap1, a huntingtin-associated protein involved in intracellular trafficking, to regulate cerebellar and brainstem development [57]. It is known that some of these proteins play important roles in regulation of stem cell functions. These results suggest that Ahi-1/AHI-1 interacts with multiple signaling proteins and plays an important role in regulating normal brain functions by interacting with molecular partners in tissue- and disease-specific manners.

It has been shown that expression of *Ahi-1/AHI-1* is regulated at multiple stages of hematopoiesis in a highly conserved fashion in mice and humans [17]. *Ahi-1/AHI-1* is expressed at its highest level in the most primitive hematopoietic stem cells (HSC) and is rapidly down-regulated as cells differentiate. Interestingly, RNA expression of the mouse *Ahi-1* gene is 5-fold higher in the mouse hematopoietic stem cell-enriched population (Sca-1^+^lin^−^) purified from normal adult bone marrow (BM) compared to the more differentiated hematopoietic cells (lin^+^). In addition, within the different lineages of differentiated lin^+^ cells, *Ahi-1* transcript levels are 6- to 7-fold lower in the granulocyte/macrophage lineage compared with the T-lymphoid, erythroid and B-lymphoid lineages [17]. A similar pattern of down-regulated human *AHI-1* transcript levels during normal hematopoietic cell differentiation has also been observed in normal adult human BM cells, with an overall 6-fold decrease from the most primitive lin^−^CD34^+^CD38^−^ subset to the most mature lin^+^ CD34^−^ cells. In addition, similar to mouse, human lin^+^ BM cells from the granulocyte lineage showed significantly reduced expression of *AHI-1* compared to T, B and erythroid cell lineages [17]. The conserved pattern of changes in *Ahi-1/AHI-1* expression between mice and humans during multi-step hematopoietic cell differentiation suggests that *Ahi-1/AHI-1* may play important roles in the regulation of the normal hematopoietic stem cell-renewal program and downstream cell differentiation events.

### *Ahi-1/AHI-1* isoforms and mutations

*Ahi-1/AHI-1* is subject to alternative splicing and both murine and human *Ahi-1/AHI-1* genes can encode at least three isoforms (Figure 2). Notably, isoform II, the shortest *AHI-1* isoform, lacks the SH3 domain and isoform III contains additional coding sequences not present in isoform I or II [16]. Comparative genetic analysis of the evolution of the human *AHI-1* gene indicates that it has undergone positive selection during development of the human species [20]. Thus, changes in *AHI-1* are likely to have been important in the evolution of human-specific characteristics; these may include features of the mechanisms regulating early stages of normal hematopoietic cell differentiation.

Involvement of *Ahi-1* in mouse models of leukemogenesis is suggested by the high frequency of *Ahi-1* mutations observed in certain virus-induced murine lymphomas [16]. Gene-expression analyses of pre-B and T-cell leukemic cells with insertional *Ahi-1* mutations has shown both increased expression of *Ahi-1* and *Ahi-1/viral* fused transcripts in the malignant cells, including deletions of the SH3 domain in some cases [16]. Recently, mutations in the human *AHI-1* gene have been found to be associated with Joubert syndrome, an autosomal recessive brain disorder [20-22]. Abnormal cerebellar development and axonal decussation were found in individuals with point mutations in *AHI-1*. These mutations generate stop codons, or amino acid substitutions, or splicing errors within the AHI-1 protein. Truncating mutations are the most frequent type (80%) that abolish completely, or partially, two critical domains of AHI-1: WD40-repeat and SH3 [22]. Altered *AHI-1* isoforms and its mutations also underline other diseases, including Joubert syndrome-associated nephronophthisis and autism, and metabolic syndromes [18, 19, 22, 24-27]. Therefore, it is very likely that truncated forms of AHI-1 are critical in development of diseases such as Joubert syndrome and specific types of human leukemia.

## BIOLOGICAL FUNCTIONS OF *AHI-1* IN HUMAN T-CELL LYMPHOMAS

### Deregulated expression of *AHI-1* in human cutaneous T-cell lymphoma cells

The first evidence that *AHI-1* may be involved in the regulation of human leukemia and lymphoma development is based on an interesting observation that *AHI-1* transcripts are significantly higher in a broad spectrum of established human leukemic and lymphoid cell lines, compared to normal human BM. The highest expression level is in two cutaneous T-cell lymphoma (CTCL) cell lines, Hut78, derived from a blood sample of a patient with Sezary Syndrome (SS), and Hut102, derived from the blood of a patient with mycosis fungoides (MF), where increases in *AHI-1* transcripts of 40-fold have been detected compared to normal BM [17]. CTCL is a heterogeneous group of T-cell lymphomas that are characterized by malignant T-cells that infiltrate the skin. SS and MF are two major subtypes of CTCL which together account for more than 70% of all CTCL cases [61-64]. SS is the leukemic form of CTCL and is characterized by erythroderma, generalized lymphadenopathy and the presence of malignant mature memory T-helper cells (CD4^+^CD7^−^CD45RO^+^), called Sezary cells, in the skin, lymph nodes and peripheral blood [64-66]. *AHI-1* is expressed at significantly higher levels at both RNA and protein levels in primary Sezary cells (CD4^+^CD7^−^) from patients with SS compared to normal CD4^+^ T-cells from normal controls [18, 24]. Particularly, *AHI-1* isoform II, lacking the SH3 domain, shows the highest expression in SS samples compared to controls [18, 24]. Little is known about the molecular pathways involved in the development of CTCL; however, the marked deregulation of *AHI-1* in CTCL cell lines and primary Sezary cells suggests a potential oncogenic role for *AHI-1* in this group of diseases.

### Evidence of an oncogenic role of *AHI-1* in CTCL cells

**Figure 3:**
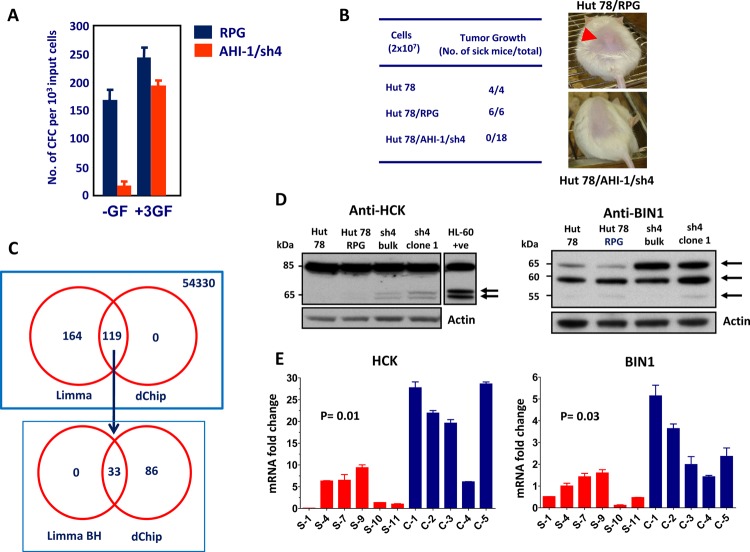
Biological effects of *AHI-1* suppression in Hut78 cells and identification of its potential cooperating genes (A) The number of CFCs able to make colonies in the absence of any growth factor (-GF) is significantly lower in Hut78 cells with *AHI-1* suppression (AHI-1/sh4) compared to the empty vector control (RPG). This effect is rescued by adding the three growth factors (IL-2, IL-4 and TNF-α) to the media. (B) Subcutaneous injection of NOD/SCID-β2m immunodeficient mice with Hut78 cells and empty vector controls (Hut78/RPG) results in tumor formation within 4 weeks in all injected mice. *AHI-1*-suppressed Hut78 cells cannot form any local tumors in these mice even after 20 weeks. (C) Venn diagram of differentially expressed genes from the microarray analysis selected by both Limma and dChip analyses. Affymetrix GeneChip microarray analysis identified several differentially expressed genes in *AHI-1*-suppressed cells compared to Hut78 and empty vector controls. Initial Limma analysis selected 283 differentially expressed probe sets, which was further refined to 33 with the Benjamini and Hochberg (BH) P value adjustment. (D) Protein expression of HCK (left panel) and BIN1 (right panel) in *AHI-1*-suppressed cells (sh4-bulk and sh4-clone1) compared to Hut78 and Hut78/RPG controls. The two isoforms of HCK and the 65 kDa isoform of BIN1 show upregulation in the absence of *AHI-1*. (E) The mRNA expression levels of *HCK* (left panel) and *BIN1* (right panel) are significantly downregulated in six SS patients (red bars) compared to five CD4^+^ T cell samples from normal controls (blue bars).

To obtain direct evidence that deregulated expression of *AHI-1* contributes to the transformed properties of human CTCL cells, knockdown of *AHI-1* expression in Hut78 cells was performed using retroviral-mediated RNA interference (RNAi). A screen of nine constructs that produce short hairpin *AHI-1* transcripts yielded one that specifically inhibited *AHI-1* expression in transduced Hut78 cells by 80%, as evaluated by quantitative real-time RT-PCR (Q-RT-PCR), Northern and Western blot analyses [18, 24]. Hut78 cells are characterized by several interesting transforming properties, including autocrine production of Interleukin (IL)-2, IL-4 and tumor necrosis factor-alpha (TNF-alpha), growth factor independence and the ability to produce tumors in mice [67-70]. Interestingly, retroviral-mediated suppression of *AHI-1* reduced autocrine production of IL-2, IL-4 and TNF-alpha in Hut78 cells by up to 85% and caused a significant reduction in their growth factor independence in semi-solid cultures (up to 10-fold) and in single-cell cultures (4-fold) by comparison to cells transduced with a control vector. It was interesting to note that these phenotypes can be restored *in vitro* in the presence of all three growth factors or IL-4 and TNF-alpha alone, but not IL-2 alone, indicating that *AHI-1* expression is important in mediating autocrine production of cytokines that may have a pathogenic role in the progression of disease (Figure 3A). In addition, aberrant expression of IL-2 and TNF-alpha also occurs in primary CD4^+^CD7^−^ Sezary cells, further supporting the idea that a multi-factorial autocrine mechanism mediated by *AHI-1* could be involved in disease development. Importantly, the ability of Hut78 cells to produce tumors in NOD/SCID-beta2microglobulin^−/−^ mice within 4 weeks was also lost when *AHI-1* expression was suppressed (Figure 3B) [18]. Thus, lymphomagenic activity of Hut78 cells is somehow dependent on the expression of *AHI-1*. Taken together, these findings provide strong evidence of the oncogenic activity of *AHI-1* in human T-cell lymphomagenesis; its deregulation may contribute to the development of human CTCL, including Sezary syndrome. Further studies will be needed to fully understand the molecular mechanisms, biological functions and clinical role of deregulated *AHI-1* expression in CTCL.

### Identification of BIN1 and HCK as potential mediators of *AHI-1* in CTCL

Microarray analysis using the Affymetrix Human Genome U133 plus 2.0 Arrays which contains over 47,000 transcripts (54,330 probes) recently identified several differentially expressed genes that may play critical roles in *AHI-1*-mediated leukemic transformation of human CTCL cells (Figure 3C) [24]. Two strong candidates identified in this study are a tyrosine kinase, HCK, and a tumor suppressor, *BIN1*, which show upregulation at both RNA and protein levels in *AHI-1*-suppressed CTCL cells (Figure 3D) [24]. HCK (hematopoietic cell kinase) is a member of the Src family tyrosine kinases and its expression is restricted to hematopoietic cells with predominant expression in myeloid lineage cells and B lymphocytes [71]. It has been reported that *HCK* has oncogenic potential in Philadelphia chromosome-positive (Ph^+^) leukemia and lymphoma cells [71, 72], however, other studies also demonstrated tumor suppressor functions for *HCK* in Ph^−^ leukemias [73-75]. In CTCL cells, changes in HCK protein expression and its phosphorylation were observed in *AHI-1*-suppressed or overexpressed cells, and suppression of activities of Src family kinases, including HCK, by TKI (dasatinib) treatment resulted in reduced or increased growth factor-independent growth of *AHI-1*-overexpressed or -suppressed cells in a dose-dependent fashion [24]. These results thus suggest that HCK could be a critical player and potential target in *AHI-1*-mediated CTCL cell transformation.

**Figure 4:**
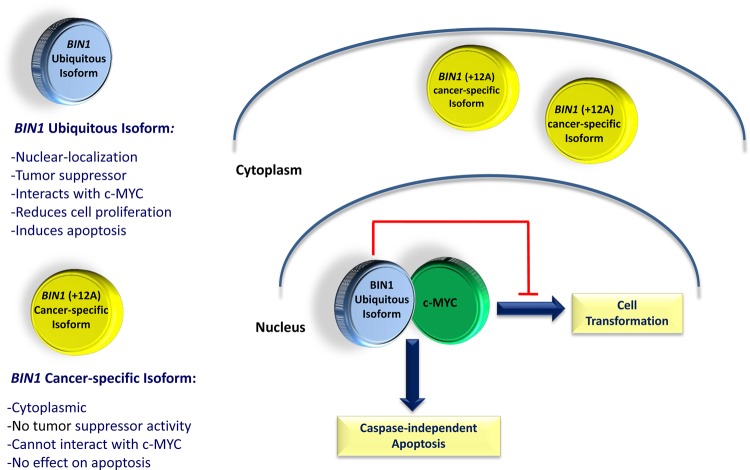
Schematic diagram of ubiquitous and cancer-specific isoforms of *BIN1*, their cellular localization and biological functions Ubiquitous isoform of *BIN1* is localized in the cell nucleus. It can physically interact with the c-MYC oncogene, inhibiting its transforming activity and increasing the caspase-independent apoptosis in cancerous cells. The cancer-specific isoform of *BIN1*, with the inclusion of exon 12A, is localized in the cytoplasm, cannot interact with c-MYC and does not have any tumor suppressor activity in cancerous cells.

*BIN1* (bridging integrator 1) is a nucleocytoplasmic adaptor protein that was first identified through its interaction with MYC oncoprotein, where it inhibits its transforming activity [76]. MYC is involved in the development of many cancers, where its overexpression is associated with poor prognosis. *BIN1* attenuation is frequently described in several cancers, including lung, breast and prostate cancer [77-80]. Alternative splicing can yield more than 10 isoforms of *BIN1* with diverse patterns of distribution between tissues, subcellular localization and protein interactions [76, 78, 81, 82]. Notably, only nuclear-localizing isoforms of *BIN1* have tumor suppressor activities that can restrict proliferation, survival, and immune escape of oncogenically transformed cells (Figure 4) [24, 83-85]. In particular, aberrant splicing of a brain-specific exon (exon 12A, Figure 4) in malignant cells can abolish the tumor-suppressor activity of BIN1 by interfering with MYC binding [77], which is regulated by phosphorylation of MYC at Ser62 [86]. It has recently been documented that *Bin1* loss can promote immune escape in cancer by deregulating the immunomodulatory enzyme indoleamine 2, 3-dioxygenase (IDO); IDO inhibitors have also been found to potentiate cancer chemotherapy [87]. Moreover, it has been shown that Bin1 interacts with the c-ABL tyrosine kinase in an SH3-dependent manner [88]. Similarly, AHI-1 has recently been found to physically interact with BCR-ABL to mediate malignant transformation of CML stem/progenitor cells [19]. Interestingly, characterization of *BIN1* in normal and leukemic hematopoietic cells has not been previously described; nevertheless, induced up-regulation of *BIN1* is observed in *AHI-1* suppressed cells and down-regulation of *BIN1* is found in SS patient samples (Figure 3D &E). Knockdown of *AHI-1* expression in CTCL cells can normalize their transforming activity and this effect seems to be associated with induction of up-regulation of *BIN1* and down-regulation of its interacting oncoprotein, MYC [24]. Thus *AHI-1* may directly or indirectly inhibit expression of *BIN1* to enhance its oncogenic activity, possibly through interaction with MYC. These findings strongly suggest that *AHI-1* may cooperate with *BIN1* in the loss of its tumor suppressor activity through its interacting oncoprotein MYC to mediate cellular proliferation and apoptosis control of human CTCL cells and drive human CTCL pathogenesis.

## BIOLOGICAL FUNCTIONS OF *AHI-1* IN HUMAN CHRONIC MYELOID LEUKEMIA

### Clinical challenges in the treatment of CML

CML has long served as a paradigm for generating new insights into the cellular origin, pathogenesis and improved treatment approaches for many types of human cancer. It is a clonal, multi-step, multi-lineage myeloproliferative disease that typically evolves through three distinct stages: chronic phase (CP), accelerated phases (AP) and blast crisis (BC) [1, 2]. The feature that uniquely defines CML is a clone-specific *BCR-ABL* fusion gene that encodes an oncoprotein with constitutively elevated tyrosine kinase (TK) activity, driving the pathogenesis of the disease [4-6]. Activation of BCR-ABL deregulates cellular proliferation, apoptosis, and genomic stability of primitive CML cells through effects on multiple intracellular signaling pathways such as the JAK2-STAT, RAS and phosphatidylinositol 3-kinase (PI3K) pathways [5, 6]. Recognition of the consistent molecular and genetic alteration of *BCR-ABL* in CML patients' leukemic cells led to the development of TKIs with selectivity for the BCR-ABL kinase [4, 89, 90]. Imatinib (IM) was the first TKI developed and has proven effective for treatment of early phase CML [8-10]. However, early relapses, acquired drug resistance and persistence of leukemic stem cells remain significant issues in many CML patients [10, 91, 92]. The major clinical limitations encountered with IM therapy are: (1) IM must be given continually for many years and its discontinuation always results in rapid reappearance of large numbers of leukemic cells [93-96]; (2) Even in treated patients, 15-25% of patients in early CP and up 40% with AP disease will fail treatment, indicating a need for alternatives [10, 92, 97, 98]; and (3) Relapses are frequently associated with mutations in the BCR-ABL kinase domain [91, 92, 99]. Dasatinib (DA) and Nilotinib (NL) are the second generation of TKIs [89, 90]. Initial clinical experience with both DA and NL indicates that they may also elicit inadequate responses and fail to prevent early disease progression in some patients [100, 101]. In particular, the T315I mutation, found in patients with IM-resistant disease, has been shown to also mediate resistance to DA and NL [102, 103]. Clinical evidence thus suggests that single agent molecularly-targeted therapy may not cure most patients as molecular remissions are rare. These observations emphasize the need for development of new agents and new strategies to prevent continuous development of resistant subclones in primitive CML cells.

### Distinct features of CML stem cells resulting in drug resistance

**Figure 5:**
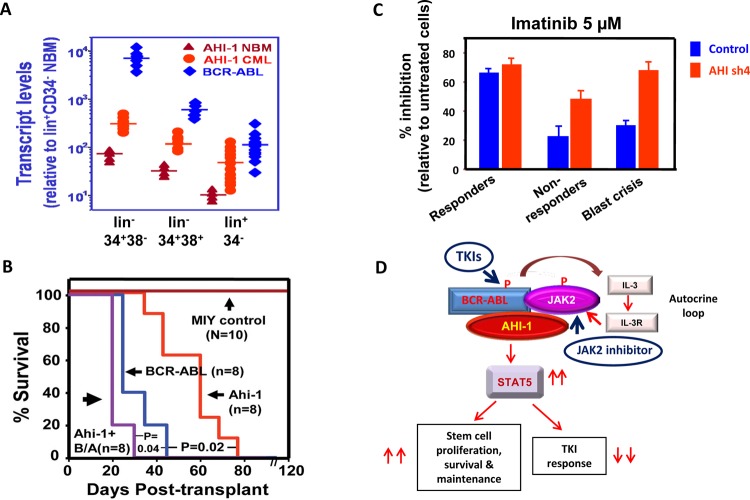
Model of the targeting of the AHI-1-BCR-ABL-JAK2 complex in CML stem cells by combination treatment with TKI and JAK2 inhibitors (A) Detection of increased transcript levels of *AHI-1* and *BCR-ABL* in a CML stem cell-enriched population (lin^−^CD34^+^CD38^−^), progenitor cells (lin^−^CD34^+^CD38^+^) and their differentiated cells (lin^+^CD34^−^) as compared to purified normal bone marrow cells using Q-RT-PCR. (B) Overexpression of *Ahi-1* induces a lethal leukemia *in vivo* and enhances the effects of *BCR-ABL*. Survival curves of NOD/SCID-β2M^−/−^ mice injected with BaF3 cells transduced with control vector, *Ahi-1*, *BCR-ABL* and *Ahi-1* plus *BCR-ABL* (B/A). (C) Inhibition of colony formation in semi-solid media by Imatinib in lin^−^CD34^+^ CML progenitor cells from IM-responders, non-responders and blast crisis patient samples transduced with either a control vector or AHI-1/sh4 vector with suppression of *AHI-1*. (D) Schematic diagram of the AHI-1-BCR-ABL-JAK2 interaction complex that regulates constitutive activation of BCR-ABL and JAK2/STAT5 and results in increased proliferation, survival and a reduced TKI response in CML stem and progenitor cells. Targeting both BCR-ABL and JAK2 activities to destabilize this protein interaction complex may present a potential therapeutic option for CML.

Accumulating evidence indicates that primitive quiescent CML cells are relatively unresponsive to TKIs [104, 105]. We and others have recently discovered that CML stem cells are insensitive to IM, with multiple unique features that would be expected to promote intrinsic and acquired resistance to BCR-ABL-targeted therapeutics [2, 106-109]. These include elevated *BCR-ABL* expression and TK activity (Figure 5A), deregulated expression of several transporter genes (*OCT1, ABCB1* and *ABCG2*) and a high degree of genetic instability. Thus, leukemic stem cells are a critical source of disease recurrence and a significant reservoir for the emergence of drug-resistant subclones and it is therefore critical to identify other therapies targeting CML stem cells to overcome resistance.

### Deregulated *AHI-1* expression in CML stem cells and its enhanced transforming activity in conjunction with *BCR-ABL in vitro* and *in vivo*

Interestingly, overexpression of either *Ahi-1* or *BCR-ABL* alone in a murine IL-3 dependent pro-B cell line, BaF3, increases proliferation and viability of these cells in the absence and presence of IL-3. However, overexpression of both of these genes simultaneously enhances these effects [19]. Moreover, intravenous injection of NOD/SCID-beta2m immunodeficient mice with either *Ahi-1*- or *BCR-ABL*-transduced BaF3 cells causes lethal leukemia within 70 and 40 days, respectively (Figure 5B). However, leukemogenic activities of co-transduced *Ahi-1* and *BCR-ABL* are further enhanced, producing a shorter latency of 26 days [19]. In addition, overexpression of either *Ahi-1* or *BCR-ABL* alone in primitive murine hematopoietic stem cells (HSC) can increase their proliferation in liquid media, as well as elevating the number of colonies from both the colony forming cell (CFC) assay, an assay used to measure the activity of progenitor cells, and the long term culture-initiating cell (LTC-IC) assay, an assay for measuring stem cell activities *in vitro* [110-112]. Importantly, in murine HSCs co-transduced with *Ahi-1* and *BCR-ABL*, all of these effects are enhanced [19]. Thus, overexpression of *Ahi-1/AHI-1* alone can transform primitive hematopoietic cells *in vitro* and *in vivo*; these effects are enhanced by *BCR-ABL*. In addition to overexpression studies, stable suppression of *AHI-1* by small interfering RNA in primary CML stem/progenitor cells reduces growth autonomy. Furthermore, co-expression of *Ahi-1* in *BCR-ABL* inducible cells reverses growth deficiencies exhibited by down-regulation of *BCR-ABL* and results in sustained tyrosine phosphorylation of BCR-ABL and enhanced activation of the JAK2/STAT5 pathway [19]. Importantly, co-immunoprecipitation assays have identified a new AHI-1-BCR-ABL-JAK2 interaction complex at endogenous levels in CML cells [19]. JAK2 is known to directly interact with the C-terminus of BCR-ABL and to be a critical target of BCR-ABL in CML [113-115]. Therefore, through an AHI-1-BCR-ABL-JAK2 interaction complex, AHI-1 regulates transforming activities of *BCR-ABL* transformed cells associated with sustained tyrosine phosphorylation of BCR-ABL and enhanced activation of JAK2/STAT5 (Figure 5D).

### Regulation of IM sensitivity of CML stem/progenitor cells by *AHI-1*

As described above, IM is an inhibitor of the BCR-ABL tyrosine kinase and the first-line therapy for the CML patients [4, 8, 9]. However, early relapses and IM-resistance are two major problems in some IM-treated CML patients [10]. Studies have indicated that CML stem cells, especially those that are in the quiescent G_0_ stage of the cell cycle, are less responsive to IM, and are therefore a critical target population for IM resistance [104-106]. Thus, identification of other therapies targeting CML stem cells, as well as development of complementary therapies that target molecular events downstream of *BCR-ABL* are two major challenges in the treatment of CML.

There are several close biological connections between AHI-1 and BCR-ABL. Both *AHI-1* and *BCR-ABL* transcript levels are significantly increased in the CML stem cell-enriched population (lin^−^CD34^+^CD38^−^) as compared to normal BM (Figure 5A) [17, 106, 107] and *AHI-1* and *BCR-ABL* transcript levels are significantly higher in CML stem/progenitor cells from IM-nonresponders and blast crisis patients compared to the same cells isolated from IM-responders [19]. Interestingly, the AHI-1-BCR-ABL-JAK2 complex was reported to play a key role in regulating IM response/resistance in *BCR-ABL-*transduced cells and primary CML stem/progenitors. Overexpression of *AHI-1* in human K562 cells resulted in greater resistance to IM, while suppression of *AHI-1* resulted in increased sensitivity to IM [19]. Altering AHI-1 expression in K562 cells mediates changes in phosphorylation and protein expression of BCR-ABL, JAK2 and STAT5, with enhanced activity and expression of these proteins when *AHI-1* is overexpressed, reduced activity and expression when *AHI-1* is suppressed, and restored effects in *AHI-1* suppressed cells with overexpression of *AHI-1*. Strikingly, suppression of *AHI-1* expression in primary CML stem/progenitor cells also resulted in increased TKI sensitivity (Figure 5C). Particularly, CML stem/progenitor cells from IM-nonresponders and blast crisis patients became more sensitive to TKIs when *AHI-1* expression was suppressed. Together, these findings provide strong evidence that AHI-1-BCR-ABL-JAK2 complex modulates response/resistance to TKIs in CML stem/progenitor cells (Figure 5D).

Interestingly, recent studies have demonstrated that JAK2 inhibitors (TG101209, WP1193) and a dual kinase inhibitor of JAK2 and ABL kinases (ON044580) induce apoptosis in IM-sensitive and IM-resistant CML cell lines [116, 117] and that treatment with TKIs in combination with TG101209 results in greater inhibition of CML stem and progenitor cells, compared to treatment with either TKIs or TG101209 alone or a combination of TKIs (Chen, DeGeer and Jiang, unpublished data). Thus, targeting both BCR-ABL and JAK2 in CML stem/progenitor cells will provide a rational strategy for improving the treatment outcome of CML patients (Figure 5D).

## AHI-1 IS DISRUPTED IN JOUBERT SYNDROME AND OTHER RELATED BRAIN DISORDERS

In addition to the role of *Ahi-1/AHI-1* in the development of leukemia and lymphoma in mice and humans, studies have also demonstrated its function in brain disorders such as Joubert syndrome (JS) and related disorders (JSRD), schizophrenia and autism [20, 21, 118-121]. JSRD are autosomal recessive disorders, characterized by a developmental mid-hindbrain malformation. “Molar tooth sign” is the characteristic feature on a cerebral MRI, which defines the cerebellar vermis and brainstem anomalies and is the diagnostic marker of Joubert syndrome [122-125]. JSRD is considered a multisystem disease, with extra-neurological features including retinal degeneration and cystic kidney disease (e.g. nephronophthisis) [126, 127].

Interestingly, genetic pedigree analysis has demonstrated a high linkage and association between the *AHI-1* locus and JS, a rare autosomal recessive disorder characterized by abnormal brain development and mental retardation [20, 21, 118]. A high frequency of *AHI-1* mutations can be identified in patients with JS, with most mutations being frameshift or nonsense mutations which result in truncated N-terminal *AHI-1* or loss of the WD40-repeat and/or SH3 domains [20, 21]. Recently, several linkage and association studies have further identified *AHI-1* as a susceptibility gene for schizophrenia, a major neuropsychiatric disorders associated with depression [119-121, 128, 129]. It is worth noting that several features of autism spectrum disorder (ASD), such as deficits in social behaviour, language dysfunction and repetitive behaviours, have also been described in up to 40% of patients with JS [130, 131]. A three-stage family-based association study has demonstrated evidence of an associated haplotype in *AHI-1* with ASD in a region of the genes that has also been associated with schizophrenia [25]. These findings suggest an important role for *AHI1* in common brain disorders affecting human cognition and behaviour.

Despite a strong genetic association of the *AHI-1* gene with susceptibility to several neuronal diseases, the functions of *AHI-1* in regulating normal brain development and disease pathogenesis remain largely unknown. It has recently been reported that Ahi-1 forms a stable complex with huntingtin-associated protein 1 (Hap1) in mouse brains [57], a protein which is important for neonatal development and involved in intracellular trafficking [23, 132-135]. Ahi-1 and Hap1 stabilize each other; *Hap1* knockout mice display a significant reduction in Ahi-1 expression levels, defective cerebellar development and abnormal axonal decussation. Similarly, suppression of *Ahi-1* in cerebellar neurons from postnatal mouse brains decreases Hap1 levels. In addition, truncated Ahi-1, which corresponds to the mutations in JS, prevents neurite outgrowth in neuronal culture and is unable to stabilize Hap1 [57]. Furthermore, reduction in either *Ahi-1* or *Hap1* in cerebellar neurons from postnatal mouse brains reduces the protein level of tyrosine kinase receptor B (TrkB) [57], which is critical for neuronal differentiation and brain development [136, 137]. Similarly, *Ahi-1* deficiency in mouse brain alters TrkB signaling by promoting the degradation of endocytic TrkB, reducing TrkB signaling in neuronal cells and resulting in depressive phenotypes, which can be alleviated with antidepressant drugs or by overexpression of TrkB in the amygdala [58]. These results provide evidence for the involvement of *Ahi-1* deficiency in depression, which occurs in JSRD and has been found to be associated with the *AHI-1* gene locus.

JSRD has additional neurological features, such as nephronophthisis and retinal degeneration [126, 127]. *Ahi-1*-null mice with complete loss of the Ahi-1 protein have normal embryonic development; however, the mice show postnatal runting and the majority do not survive to adulthood [59, 138]. The brain morphology of these mice is highly preserved, suggesting that other effects outside of the nervous system influence the survival of these mice [138]. One study has demonstrated the development of the cystic kidney disease nephronophthisis as a potential cause of death in *Ahi-1*-null mice [59]. Furthermore, mouse models with conditional *Ahi-1* knockout in the kidneys have demonstrated a significant decrease in basal Wnt activity [59]. The Wnt signalling pathway functions in a broad array of cellular processes, and mutations in this pathway have been identified in variety of diseases, from developmental disorders to cancer [139, 140]. In addition, Wnt activity is upregulated in mouse renal injury, suggesting its potential role in adult renal homeostatic injury repair [141]. Interestingly, Ahi-1 interacts with beta-catenin, an integral component in the Wnt signaling pathway, and facilitates its translocation and accumulation in the nucleus, resulting in positive modulation of downstream transcription [59]. *In vivo* studies have shown that *Ahi-1* is also required for the Wnt response to injury and renal tubule repair, a function that is abrogated in *Ahi-1*-knockout kidneys, leading to renal cystogenesis [59]. Moreover, other studies have demonstrated that *Ahi-1* knockout mice fail to form photoreceptor sensory cilia and photoreceptor outer segments. The retinal degeneration in *Ahi-1* knockout mice resembles the retinal phenotype observed in patients with JSRD [138, 142]. In summary, the use of *Ahi-1-*deficient mouse models has facilitated our understanding of the molecular mechanisms and the pathogenesis of JS and its related JSRD, and enabled identification of potential Ahi-1/AHI-1 interacting proteins critical in the development of these diseases.

## CONCLUSION

The discovery of ABL tyrosine kinase inhibitors has marked a major advance in the cancer therapy field. It has provided an excellent example of how a specific molecular abnormality can be targeted therapeutically to transform a life-threatening malignancy into a chronic disease. However, TKI therapy is not curative and does not eliminate leukemia stem cells, which remain a potential of relapse. Resistance to single TKI therapy is increasingly being recognized, and more effective strategies using combination therapies are needed to combat the emergence of disease resistance by effectively eradicating leukemic stem cells. Understanding the unique biological properties of cancer stem cells and identifying oncogenes and tumor suppressors and the molecular networks that regulate these properties continue to provide new insights into the complex processes of malignant transformation and disease progression and to uncover improved therapeutic options to eradicate these critical cells and develop molecular cures. Additional insights of broader applicability and benefit will also be forthcoming from such studies.
